# VLPs Derived from the CCMV Plant Virus Can Directly Transfect and Deliver Heterologous Genes for Translation into Mammalian Cells

**DOI:** 10.1155/2019/4630891

**Published:** 2019-10-29

**Authors:** María V. Villagrana-Escareño, Elizabeth Reynaga-Hernández, Othir G. Galicia-Cruz, Ana L. Durán-Meza, Viridiana De la Cruz-González, Carmen Y. Hernández-Carballo, Jaime Ruíz-García

**Affiliations:** ^1^Physical Biology Laboratory, Institute of Physics, Autonomous University of San Luis Potosí, San Luis Potosí, Mexico; ^2^Analytical Pharmacology Laboratory, Faculty of Medicine, Autonomous University of San Luis Potosí, San Luis Potosí, Mexico; ^3^Biophysics Laboratory, Institute of Physics, Autonomous University of San Luis Potosí, San Luis Potosí, Mexico

## Abstract

Virus-like particles (VLPs) are being used for therapeutic developments such as vaccines and drug nanocarriers. Among these, plant virus capsids are gaining interest for the formation of VLPs because they can be safely handled and are noncytotoxic. A paradigm in virology, however, is that plant viruses cannot transfect and deliver directly their genetic material or other cargos into mammalian cells. In this work, we prepared VLPs with the CCMV capsid and the mRNA-EGFP as a cargo and reporter gene. We show, for the first time, that these plant virus-based VLPs are capable of directly transfecting different eukaryotic cell lines, without the aid of any transfecting adjuvant, and delivering their nucleic acid for translation as observed by the presence of fluorescent protein. Our results show that the CCMV capsid is a good noncytotoxic container for genome delivery into mammalian cells.

## 1. Introduction

For many years, animal viruses have been used as vectors for gene delivery and gene therapy purposes. More recently, because of their relative structural and chemical stability, easy production, lack of toxicity, and pathogenicity in animals or humans, plant viruses and bacteriophages are increasingly being used in nanobiotechnology for the same purposes [1].

Most plants viruses are formed only by the capsid protein (CP) and its corresponding genetic material, and because they lack a membrane envelope, they are termed as nonenveloped or naked viruses [2]. The cowpea chlorotic mottle virus (CCMV) is a plant virus that has been widely studied; it is a naked virus, and its capsid is made of 180 identical protein units. The viral genome consists of four types of positive single-stranded RNAs, which are packaged in three structurally similar capsids. RNA1 is 3171 nt long, while RNA2 is 2774 nt long; they are packaged in individual capsids. RNA3 and RNA4 consist of 2173 and 824 nucleotides, respectively [3], and are copackaged in a single capsid. CCMV has the ability to self-assemble *in vitro* from its components to form infectious virions [4] or even to form empty capsids without its genomic material [5]. It has been demonstrated that its capsid is capable of encapsidating both biological [6–8] and nonbiological materials [9–14] That is, when the capsid carries cargos different from its genetic material, it is called a virus-like particle (VLP). These cargos can be different polyanions, such as charged polystyrene sulphonate (PSS) [11], mineralized salts [9], negatively charged nanocolloidal particles [10], nanolipospheres [14], chromophores [12], and enzymes [13], among other cargos.

Studies carried out by Mukherjee et al. [15] have shown the use of the CCMV CP as a nanocontainer for the encapsidation of short dsDNA. However, it was found the formation of tubular capsids rather than the native spherical shape; they found very stable structures with uniform diameters but with different lengths. They suggested that the protein starts self-assembling with the formation of a hemispherical cap, and from it, the protein begins self-assembling into tubular structures along the DNA, closing the tube on the other side with another hemispherical cap. In other studies, they have also used dsDNA for encapsidation observing again the formation of tubular structures [16]. Therefore, it seems that the CCMV CP prefers to form tubular structures due to the large persistence length of the DNA molecules and the small size of the spherical capsid. However, when they used the CP from the human hepatitis B virus, a much larger capsid than the CCMV capsid, for the encapsidation of DNA with different lengths, they observed the formation of spherical structures, similar in size to the that of hepatitis B virus. They encapsidated dsDNA of 600 and 1600 bp and ssDNA of 3000 nt. However, they reported that the best spherical structure was formed when the ssDNA was used. That is, for the 600 bp dsDNA, they found incomplete structures that they named aberrant structures, which are complexes of incomplete capsids. For the 1600 bp dsDNA, they observed the formation of even larger complexes or clusters of capsids [17].

Furthermore, Cadena-Nava et al. [18] have used the CCMV CP for the encapsidation of ssRNA from BMV and Sindbis viruses as well as noncoding RNAs of different lengths, ranging from 140 to 12000 nt. They showed that depending on the RNA length, one capsid can contain various short RNA molecules, or in the case of long RNA molecules, they could require up to four capsids to be packaged. In addition, the assembly studies showed that RNAs of different lengths can be completely packaged as long as the protein/RNA weight ratio is sufficiently high, and in all cases, the optimal assembly weight ratio of protein to RNA was 6 : 1, independent of the length of the RNA.

Viruses use different mechanisms to introduce their genetic material inside different cells [19]. Briefly, most viruses must first recognize and bind onto the cells they infect, mainly throughout receptors that are present on the exterior of the host cells. This binding is the first step for virus introduction into the cells, e.g., by receptor-mediated endocytosis. A naked or nonenveloped virus penetrates by endosome membrane lysis or pore formation [2]. Then, the nucleic acid is released into the cell and begins the viral component synthesis using the host cell machinery for the replication process. The life cycle of a bacteriophage begins with the attachment of special tail fibers to the cell wall, followed by the injection of its nucleic acid [20].

In the case of a plant virus cycle, they are often spread from plant to plant by vector organisms such as insects, but in some cases, some fungi, nematode worms, and single-celled organisms can carry out this function [21]. For virus reproduction, and thereby establishing infection, it must enter the host cells and use those cell materials for replication. Once replication occurs, the virus goes through the plasmodesmata conducts that connect other cells to start new infections. The mechanisms of packing are not well understood, but it is suggested that the CP, in some cases, can be distinguished between different RNAs, and in other cases, the CP seems to be an RNA packaging signal located in the genome, in a segment sterically accessible as a domain to be recognized to be assembled [22].

In general, because of their simplicity and relative size with respect to cells, viruses have become the masters for the delivery of genetic material into cells. Different research groups are taking advantage of these characteristics to use viral capsids as nanovehicles to introduce different cargos into mammalian cells. Most viral capsids used for gene therapy, vaccines, etc., come so far from animal viruses; for example, retrovirus [23, 24], adenovirus [25], or adeno-associated viruses [26]. However, there are always safety concerns on the use of animal viral capsids [27–29]. An alternative strategy is the use of capsids obtained from plant viruses, whose capsid protein (CP) has been shown to be biocompatible with mammalian cells, e.g., CCMV K [30], cowpea mosaic virus (CPMV) [31], and brome mosaic virus (BMV) [32]. In fact, plant viruses have shown a great potential for medical applications [33, 34]. For example, Destito et al. [35] used the CPMV, a virus structurally similar to CCMV, whose surface was modified by functionalizing it with a conjugated PEG-folic acid (FA) that binds to the overexpressed FA receptors on cancer cells. Furthermore, the same group later showed that CPMV could be able to enter macrophages and HeLa cells through caveolae-mediated endocytosis and micropinocytosis [36]. In these experiments, the external surface of the virus was altered with a fluorescence probe and lipids. In addition, they used a huge MOI (multiplicity of infection) of 1 × 10^6^ CPMV particles per cell. Furthermore, these experiments do not show that the virus can deliver nucleic acids for translation. Another important work in the use of plant viruses for medical applications is that done by the Gelbart/Knobler group [37]. They used CCMV-based VLPs for the delivery of a replicon from EYFP and Sindbis viruses into BHK cells. They show that the VLPs can deliver the RNA to be translated into fluorescence protein. In addition, they added a replicon (with a MOI-like value > 100) to the cells in order to supply the replication machinery necessary for EYFP production. Furthermore, they use the adjuvant lipofectamine to aid the entrance of the VLPs into the cells, which it has some degree of toxicity [38]. On the one hand, Wu et al. have also modified the surface capsid of CPMV with a highly reactive linker, which allows the bioconjugation of a H5 polyarginine peptide in HeLa cells and depending on the density of H5 peptides makes the uptake of the CPMV capside into the cells easier than those without modification [39]. On the other hand, others use the CCMV and BMV as a nanocontainer propelled by hydrogen peroxide, without the possibility of using this approach for medical applications [40]. Recently, VLPs made of CCMV capsid were modified on the surface for the delivery of siRNA; this modification consisted in the use of the linker M-lycotoxin peptide L17E and PEG. In addition, they have used lipofectamine as a control for the delivery of the VLPs [8].

In this work, we show that VLPs formed by plant virus capsids are able to enter mammalian cells by themselves, without the aid of any adjuvant or surface modification on the capsid, and then, the VLP's do their function inside the cell. Here, we use VLPs based on the CCMV plant virus capsid and show for the first time that they are capable of transfecting *in vitro* directly into mammalian cells HEK293, HeLa, or HK2 and releasing a heterologous nucleic acid. In this case, an mRNA that codes for the enhanced green fluorescent protein (EGFP) was used as a reporter gene. This CCMV capsid approach can be used for gene therapy and drug applications to release negative nanocargos in mammalian cells, avoiding the use of toxic material for the cells.

## 2. Results

### 2.1. Nucleic Acid Production

**Figure 1:**
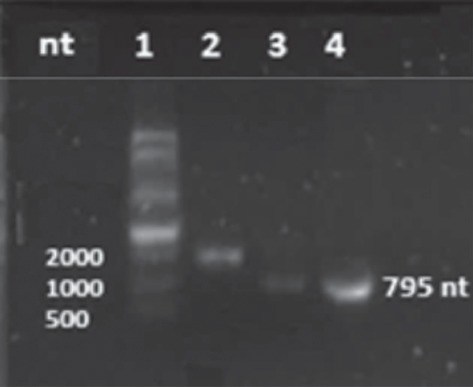
mRNA-EGFP transcription. Capped (lane 3) and uncapped (lane 4) mRNAs that code for EGFP were synthesized along with a luciferase mRNA (control, lane 2). Their integrity was visually evaluated using electrophoresis agarose gels stained with GelRed™. Transcripts are compared with the ssRNA ladder (lane 1). Single gel experiment without cropping.

Capping of mRNA protects it against degradation. Because the assembly of mRNA into VLPs can provide an additional level of protection, we decided to test if uncapped EGFP mRNA delivered by VLPs can actually survive after transfection and uncoating and be translated. Thus, both capped and uncapped EGFP mRNAs were prepared. The purity and integrity of the mRNA molecules were evaluated by UV absorbance ratio A260/A280 and agarose gel electrophoresis, respectively. The A260/A280 ratio was 2.0 for both the capped and uncapped RNA molecules.  Figure 1 shows both capped (lane 3) and uncapped (lane 4) EGFP mRNAs as well as an mRNA that codes for enzyme luciferase, used here as control for transcription reaction (1800 nt, lane 2). As can be seen, no degradation was found, and therefore, all mRNAs were adequate to be encapsidated by the CCMV capsid protein.

### 2.2. Structural Characterization of VLPs

#### 2.2.1. Retardation Gel

**Figure 2:**
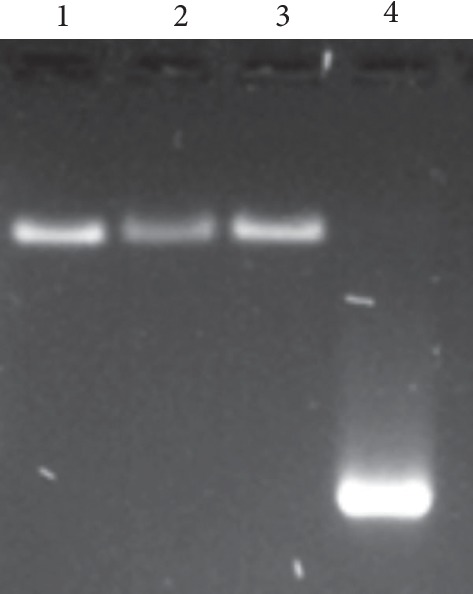
VLPs containing mRNAs in a mass ratio of 6 : 1 (CP : mRNA). VLPs were run in electrophoretic shift assay. For comparison, 0.2 *μ*g of wt-CCMV virus (lane 1) were used as control. The electrophoretic mobility of VLPs containing the capped and uncapped transcript for the EGFP is shown in lanes 2 and 3, respectively. The mRNA transcript for EGFP is shown in lane 4. The gel was stained with GelRed™, which also stains the mRNA inside the capsids, which shows that there are no free mRNA in the lanes of the VLPs. Single gel experiment without cropping.

Synthetic mRNAs coding for EGFP were then assembled into CCMV VLPs, and to determine if the mRNA was encapsidated by the CCMV capsid protein, we performed retardation gel assays.  Figure 2 shows VLPs containing both capped and uncapped mRNA molecules (lanes 2 and 3, respectively) had the same migration pattern than the wild type (wt)-CCMV assembly (lane 1). For comparison, lane 4 shows the pure EGFP mRNA transcript, which migrated faster than the VLPs and wt-CCMV, whose lanes do not show free mRNA; this experiment indicates that all mRNAs were encapsidated due to the negative charges on the exterior surface of the capsid which will interact repulsively with a nucleic acid [41, 42].

### 2.3. Analysis by TEM

**Figure 3:**
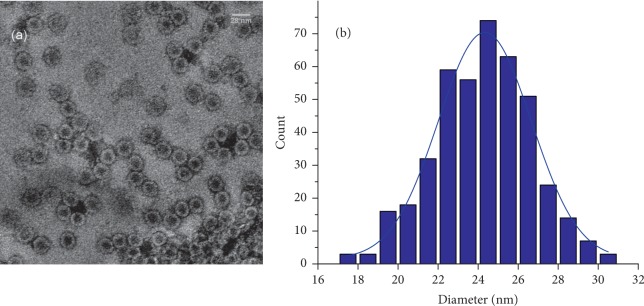
VLPs formed with the CCMV CP and containing the mRNA-EGFP (a). Negative-stained TEM image of VLPs containing capped EGFP mRNA assembled at a molar ratio CP : mRNA of 6 : 1. (b). VLPs size distribution measured in two orthogonal directions (*n* = 300 VLPs).

Direct observation of the VLPs assemblies was done by TEM.  Figure 3(a) shows VLPs formed after the encapsidation of the mRNA. Well-formed VLPs are easily observed, similar in size to the wild type CCMV, but slight differences appear after performing size distribution measurements, as shown in Figure 3(b). We found VLPs with size ranges from 18 to 30 nm with an average size of 24 nm of a total of 300 measurements. VLPs in the range of 27 to 30 nm were also observed by TEM.

### 2.4. Viability of Mammalian Cells Exposed to VLPs

**Figure 4:**
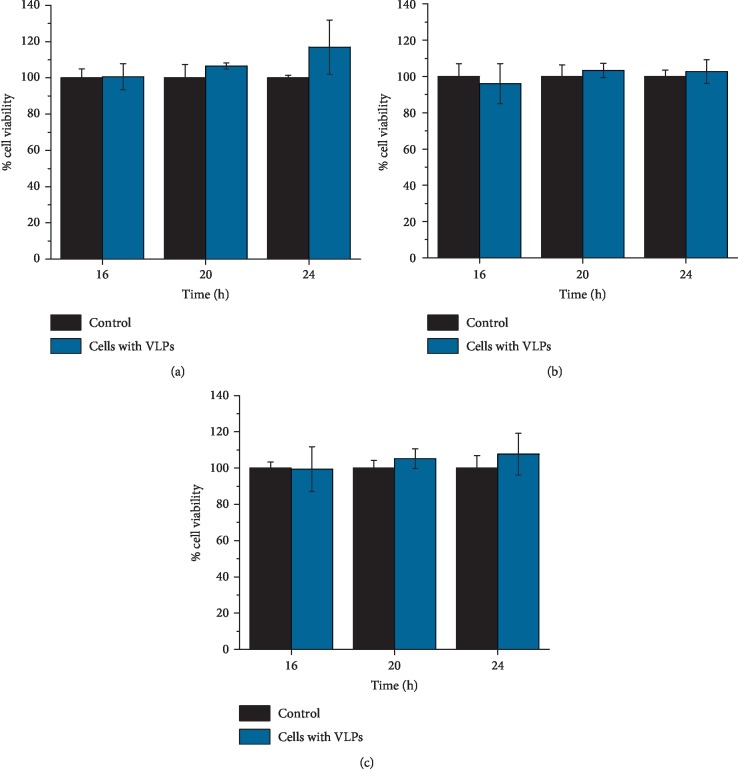
Viability of mammalian cells exposed to VLPs. Cell monolayers of HEK293 (a), HeLa (b), and HK2 (c) were incubated for 16, 20, and 24 h in the presence of 100 VLPs per cell containing capped EGFP mRNA. The effect of VLPs was compared against non-VLP-treated cultures (control). Results are expressed as relative percentage with respect to the negative control and represent the average of three independent experiments (*p* < 0.05).

Once the formation of VLPs was confirmed, three different cell lines were exposed to the VLPS: human normal kidney immortalized cells (HK2), human embryonic kidney immortalized cells (HEK293), and human epithelial adenocarcinoma cells (HeLa). First of all, the viability of VLP-exposed cells was evaluated by measuring their metabolic activity using the MTS tetrazolium salt assay (Figure 4). We found that, in comparison to the cell lines under control conditions, the viability of the HEK293 (a), HeLa (b), and HK2 (c) cell lines was not affected after 24 h of incubation with VLPs.

### 2.5. Heterologous Viral Transfection of Mammalian Cells

#### 2.5.1. EGFP Quantitation Expression

**Figure 5:**
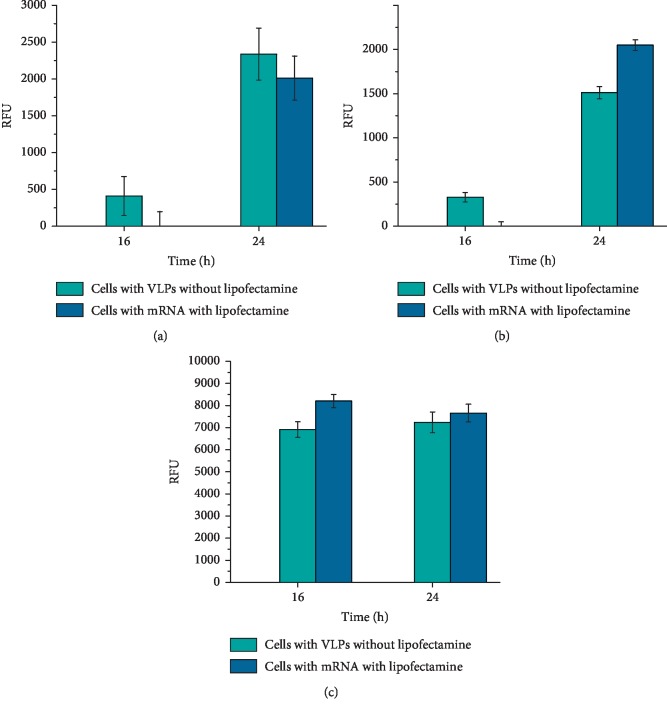
Quantification of EGFP intensity on cell monolayers. (a) HEK293, (b) HeLa, and (c) HK2 were incubated for 16 and 24 h with VLPs or mRNA. mRNA with lipofectamine and cells alone were used as positive and negative control of fluorescence, respectively. The best time for analysing the expression of EGFP was 24 h. The results are expressed as the relative fluorescence units (RFUs) in relation with the negative control.

After the viability of exposed cells was determined, we evaluated if the cargo of VLPs was properly delivered. A proper delivery by VLPs must produce fluorescence in the green region of the visible spectrum as the EGFP mRNA molecules must have been translated. We therefore evaluated the bulk fluorescence intensity of treated cells growing in monolayers by measuring the fluorescence, as shown in Figure 5. Untreated cells did not show fluorescence as expected. However, when treated cells were monitored, we detected green fluorescence. All cell lines gave good fluorescence intensity over time and also those cell lines exposed to mRNA using lipofectamine; these last results were included for fluorescence comparison with the VLPs containing mRNA without the use of lipofectamine.

### 2.6. EGFP Expression by Fluorescence Microscopy

**Figure 6:**
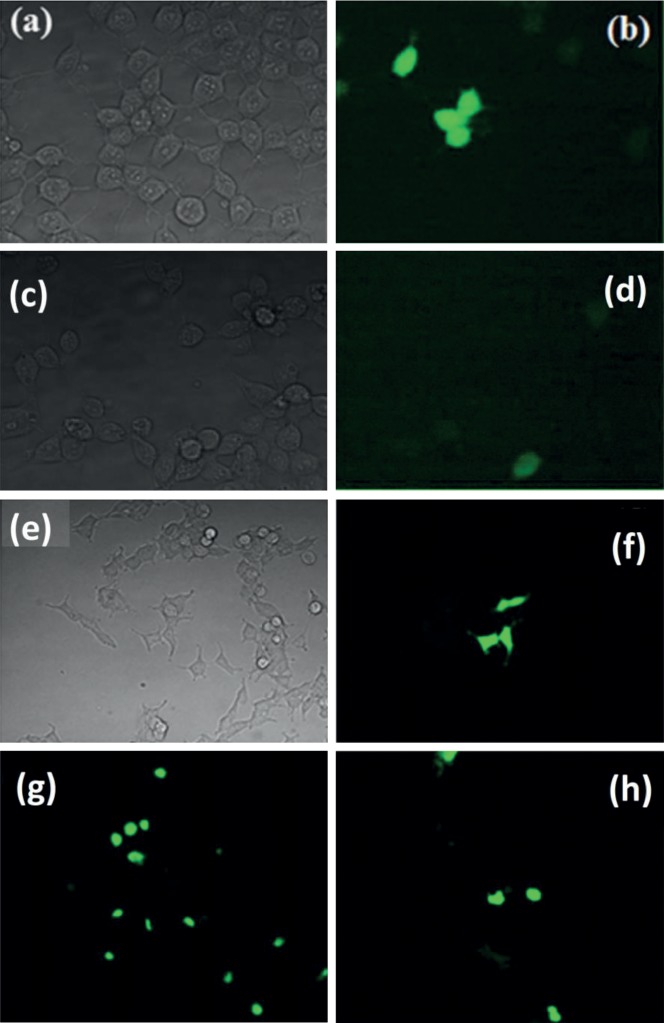
VLPs internalization in mammalian cells. HEK293 transfected with pVAX1-EGFP-DNA and mRNA with lipofectamine as controls in transmitted light and fluorescence microscopy using a 40X objective (a–d). HEK293 exposed to VLPs without lipofectamine in transmitted and fluorescence light taken with a 20X objective e and f, respectively. (g) HeLa and (h) HK2 cells exposed to VLPs show also fluorescence with a 10X objective.

The expression of EGFP within the cell lines in the presence of VLPs was also seen by fluorescence microscopy (Figure 6). First of all, the HEK293 cells were previously transfected with pVAX1-EGFP DNA and RNA with lipofectamine. They were tested to be sure that DNA and RNA were able to produce EGFP protein as observed through the appearance of fluorescence and could be used for our following experiments (Figures 6(a)–6(d)). Then, we continued with the experiments by exposing the different cell lines to the VLPs that contain the mRNA that codes for EGFP. The fluorescence started to be noticeable under a fluorescence microscope in all cell lines after 24 h of incubation of the cell lines with the VLPs-mRNA-EGFP. Figures 6(e) and 6(f) show the HEK293 in transmitted light and fluorescence microscopy, respectively. HeLa and HK2 also show fluorescence cells (g and h).

## 3. Discussion

mRNAs are unique nucleic acids for translation of different proteins; hence, they can also help us to detect successful transfections of cells as a gene reporter by producing fluorescence proteins. First, we have demonstrated that both capped and uncapped mRNAs were purified without any sign of degradation. Afterwards, we show that they can be encapsidated by the CCMV capsid protein. We tested, by a shift-electrophoretic assay, that the mRNAs were successfully encapsidated to form VLP because they migrated at the same level like the wild type CCMV. Furthermore, our experiments show that all mRNAs were encapsidated due to which no free mRNA was detected in the lanes of the VLPs in Figure 2. Another way to probe that these VLPs were well formed was by determining the size of the VLPs. We found a size distribution that goes from about 18 to 30 nm; a size of about 24 nm corresponds to a pseudo-*T* = 2 capsid, while smaller capsid sizes, about 18 nm, will correspond to *T* = 1 capsids. In addition, VLPs in the range of 27 to 30 nm will correspond to *T* = 3 capsids. The wt-CCMV capsid contains about 3000 nt long mRNAs. However, the mRNA that codes for the EGFP is made of 795 nt. Therefore, the size polydispersity of the capsids must be because VLPs contain different numbers of mRNAs molecules; that is, *T* = 1 VLPs must contain 1, *T* = 2 VLPs contain 2 or 3, and *T* = 3 contain 4 RNAs [18].

The viability studies with the VLPs indicated that the VPLs *per se* did not affect the cell metabolism and did not modify the viability of the three different cell lines evaluated, suggesting that if another cargo was included into these particles, the possible effect that could be observed in the cells exposed to VPLs could be associated to the cargo instead to the capsid protein forming the particles construct. Likewise, when the fluorescence intensity was analysed, it was worth noticing that when the HeLa and HK2 cells were exposed to VLPs, they showed lower fluorescence intensity compared with positive control experiment (we used lipofectamine to introduce mRNA with cap). It could be because not all the VLPs disassemble and expose the mRNA to ribosomes with the same efficiency for its translation [37]. It might also be that the biological affinity between the VLPs and the cells depends on the cell line type since we did observe differences on the fluorescence intensity given by the cell lines, especially we observed a higher fluorescence in the HK2 cell line than in the other two cell lines. The transfection of VLPs without the aid of any adjuvant or lipophilic agent was confirmed when the cells were seen under a fluorescence microscope demonstrating that the three different cell lines had used their cellular machinery to produce the EGFP.

On the contrary, the percentage of fluorescence with VLPs containing mRNA with or without cap showed similar results (data not shown). It is clear that, at the experimental time periods, when the fluorescent measurements were taken, there were not a lot of cells producing fluorescence. However, our results are similar to those reported by Lam and Steinmetz [8]. In addition, our experiments indicate that the nucleic acid can be used without capping, which indicates that, at least, in these conditions, the CCMV capsid was able to protect the mRNA from degradation because it carried out its translation. However, for the following experiments, we continued with cap RNA to secure the stability of mRNA.

A question remains, however, and it is related to the CCMV entry mechanism into mammalian cells, since the VLPs used here come from a naked CCMV virus and most of the viruses that infect mammalian cells contain glycoproteins for cell receptor recognition and internalization [2]; that is, they commonly have a lipid membrane with glycoproteins that recognize receptors on the cell membrane that facilitate their attachment and cell entry. Other animal viral capsids might only contain the glycoproteins attached to the capsid without a membrane, such as the adenovirus [2]. In the case of the CPMV, another plant virus, it has been determined that a 54 kDa protein called vimentin is involved in the interaction between the virus capsid and the cell surface of dendritic cells from Balb C17 and MC57 [31]. However, vimentin is an intracellular protein, localized mainly on the cytoskeleton, a surface-expressed form has recently been found on some cell types [43], and it could also be helping to the internalization of CCMV, as in the case of CPMV.

There are several strategies to deliver cargos of different materials, as was described above. However, there are some studies thus far that have demonstrated that CCMV, or any other capsid from a plant virus, when it used as nanocontainer of heterologous mRNA or other cargos, has the ability of transfecting mammalian cells. However, in these studies, the CCMV transfects with the aid of adjuvants, either lipophilic or cationic agents [37], surface modifications of the capsid [36, 44], or linkers [39]. Other studies have claimed that plant virus capsids are able to directly transfect mammalian cells by themselves. For example, Lam and Steinmetz [8] stated that the CCMV was taken up by the cells without any adjuvant. However, they did modify the capsid surface with approximately 60 Cy5 dyes per CCMV particle. Also, Tejeda-Rodríguez et al. [40] mentioned that VLP can enter by themselves. However, they used Janus viral nanomotors (JVN), which is the full virus particle with approximately half of the surface modified with a platinum layer. In other experiments, they report the mixture of BMV with tamoxifen, which is almost sure that it might not be internalized because the capsid has the viral RNA inside, and tamoxifen might be attached to the capsid surface modifying it. Furthermore, they used the fluorescent molecule NanoOrange that also might be attached to the capsid surface.

Therefore, our studies have proved that these VLPs derived from the CCMV capsid are able to directly transfect mammalian cells by themselves, since they successfully delivered their mRNA cargo and produced the EGFP protein observed by the appearance of fluorescence in HeLa, HEK293, and HK2 mammalian cell lines. In summary, we tested the hypothesis that plant virus capsids are capable of direct transfection of mammalian cells; we show that these VLPs can transfect these mammalian cells lines without the aid of any adjuvant or any other agent or even surface modification of the capsid. The VLPs have mRNA with and without cap, and we found similar results in both cases. Therefore, our results show that the VLPs also protect their cargo against RNA degradation by the RNases within the cells. Due to its nontoxicity, these VLPs particles can now be functionalized by attaching some specific ligands, through chemical modifications, that might be efficiently conjugated to CCMV capsid to reach a receptor on the target cell [35].

## 4. Conclusions

In this work, we expose three mammalian cell lines, HEK293, HK2, and HeLa cells, to our VLPs that contain an mRNA that codes for the EGFP and are formed using the plant virus CCMV capsid protein. It is assumed that plant virus capsids do not transfect eukaryotic cells, so we planned control experiments exposing the cell lines to the VLPs alone. We wanted to verify that the VLPs were not able to be up taken by the cells alone, without the aid of any adjuvant or capsid surface modification. However, to our surprise, we discovered that the VLPs were able to go through the cell membrane by themselves and delivered their gene cargo since fluorescence developed within the cells. That is, we found that these VLPs transfect the different mammalian cell lines and deliver their mRNA cargo into these cells by themselves, which was successfully translated as observed by the appearance of fluorescence due to the presence of the EGFP. The VLPs have mRNA with and without cap, and we found similar results in both cases. Therefore, our results show that the VLPs also protect their cargo against RNA degradation by the RNases within the cells. It is important to remark that our results show for the first time that a plant virus capsid is capable of transfecting mammalian cells by itself. Due to its nontoxicity, we should take advantage and use these characteristics of the CCMV capsid to deliver cargos into the cells, e.g., for gene delivery into mammalian cells for gene therapy applications. In a more general contribution, our results show that VLPs based on the CCMV capsid protein could directly translocate other types of cargos into mammalian cells, such as nanoparticles [10] or enzymes [13], which made them very attractive for other types of biomedical applications.

## 5. Methods

### 5.1. RNA Transcription

Enhanced green fluorescent protein (EGFP) was cloned into the Eco RI site of pVAX1. This plasmid (pVAX1-EGFP) was then amplified and purified using a Qiagen Maxi kit (QIAGEN, Valencia CA, USA). RNA transcription *in vitro* was carried out by RiboMAX™ large-scale RNA production systems kit (Promega, Madison WI, USA). Briefly, 50 *μ*g of pVAX1-EGFP was linearized with Xho I (NEB, Ipswich, MA, USA) at 37°C and inactivated at 65°C. Afterwards, the linearized plasmid was treated with proteinase K at 100 *μ*g/ml in 0.5% SDS, 5 mM CaCl2, and 50 mM Tris-HCl for 30 min at 37°C. Extraction of the linearized DNA with phenol : chloroform 1 : 1 and elution in nuclease-free water was carried out. 5 *μ*g of this DNA was subjected to T7 transcription reaction in 20 *μ*l with Ribo m7G Cap Analog procedure (Promega, Madison WI, USA) as indicated by the manufacturer. RNA transcript was treated with RQ1 RNase-free DNase (1 U/*μ*g) for 15 min at 37°C followed by a phenol : chloroform purification protocol and eluted in nuclease-free water. The integrity of mRNA was evaluated by denatured gel electrophoresis with formaldehyde in 1x MOPS as running buffer with diethyl pyrocarbonate (DEPC) water and stained with 0.5 *μ*l GelRed (Biotium, Fremont CA, USA). The *in vitro* transcripts were denatured for 15 min at 65°C and then kept on ice for 5 min before they were loaded onto 1.2% agarose gel. The mRNA was compared with ssRNA ladder (NEB, Ipswich, MA, USA). The purity of the mRNA samples was determined by the absorbance ratio A260/A280 and A260/A230. The purity was measured using the UV-Vis spectrophotometer (NanoDrop 2000C, ThermoScientific, MA, USA) and gave values for all samples of at least 2.0 and 2.3 for these ratios, respectively, indicating a very good purity.

### 5.2. CCMV CP Purification

Briefly, CCMV was obtained from infected cowpea plants (*Vigna unguiculata*) [4]. The leaves were sand-milled and cheesecloth-filtered to remove large debris, and then, three centrifugation processes were done to purify the virus [45]. Once the virion was purified, it was disassembled to separate the CP from its genome by dialysis of the virion in disassembly buffer [12]. Immediately, 300 *μ*l aliquots were taken and their concentration was measured under a UV-Vis spectrophotometer (NanoDrop 2000C, ThermoScientific, MA, USA). Only aliquots with purity above 1.6 (protein°:°RNA ratio) were chosen for this study. The protein was then dialyzed for 12 h against protein buffer (1 M NaCl, 20 mM Tris-HCl at pH 7.2, 1 mM EDTA at pH 8.0, 1 mM DTT, 1 mM PMSF) at pH 7.2, stored at 4°C, and used within 2 weeks.

### 5.3. Self-Assembly of CCMV CP and mRNA-EGFP for the Formation of VLPs

For the assembly reactions, a mass ratio 6 : 1 CCMV CP : mRNA EGFP were subjected to dialysis for 24 h at 4°C in assembly buffer (50 mM NaCl, 50 mM Tris-HCl, 10 mM KCl, 5 mM MgCl_2_, 1 mM DTT 0.5 mM PMSF) at pH 7.2 and dialyzed against virus suspension buffer (50 mM NaC_2_H_3_O_2_ and 8 mM Mg(CH_3_COO)_2_) for 12 h, with the pH adjusted to 4.5 with glacial acetic acid [18]. Then, the samples were returned to dialysis in the assembly buffer at pH 7.2 for at least 4 h. As a first step to evaluate the VLP formation, we proceeded to make a retardation gel in 1% agarose gel prepared in virus buffer (100 mM NaC_2_H_3_O_2_ and 1 mM EDTA at pH 5.0). 0.2 *μ*g of VLPs and CCMV samples were prepared with 30% glycerol and 10 *μ*l was loaded into the gel stained with 0.5 *μ*l GelRed (Biotium, CA, USA) and ran at 50 V for 2.5 h.

### 5.4. Electron Transmission Microscopy Analysis

The VLPs were also analysed by electron transmission microscopy (JEOL-JEM 1230, 120 keV). The samples were negatively stained with 2% uranyl acetate solution; six microliters of the sample was placed on a 300-mesh grid previously prepared with parlodium and coal covered. The sample was left to settle onto the grid surface for 1 min, and the rest was retired with a filter paper. Then, 6 *μ*l of uranyl acetate was added for 1 min onto the sample, then washed-dried with a filter paper, and the grid stored in a desiccator overnight, before they were observed under a transmission electron microscope (TEM) operated at 80 keV. Statistical data from the images were determined by the ImageJ software [46–48].

### 5.5. Cell Lines

HK2 (CRL-2190™, ATCC, Rockville, MD, USA), HeLa, and HEK293  cell lines were grown in 25 cm^2^ flasks in a 1 : 1 mixture of Dulbecco's modified Eagle medium (DMEM GIBCO-BRL® Life Tech, Thermo Fisher, MA, USA), Ham's F_12_ nutrient mixture for HK2 cells plus 10% fetal bovine serum (FBS, GIBCO-BRL®), and DMEM medium for HEK293 and HeLa cells plus 5% FBS. The medium was supplemented with 2 mM L-glutamine (Sigma-Aldrich, MO, USA), 15 mM HEPES (Sigma-Aldrich), and 5 ng/ml recombinant human epidermal growth factor (EGF, Life Technologies) only for HK2 cells (pH 7.4), and gentamicin was used for HEK293. The cultures were incubated at 37°C in a 5% CO_2_ atmosphere and 95% of relative humidity until cell monolayers were formed. The medium was replaced every 72 h, and when monolayers reached at least 80% of confluence, they were washed with a free Ca^2+^ and Mg^2+^ saline phosphate buffer (PBS: 138 mM NaCl, 3 mM KCl, 8.1 mM Na_2_HPO_4_, 1.5 mM KH_2_PO_4_, pH 7.4) and incubated with 0.125% trypsin and 0.5 mM EDTA solution for 15 min, and in some HEK293 cultures, Hanks solution was used for detaching cells. Detached cells were harvested and suspended in fresh medium at a concentration of 10^6^ cells/ml to subculture in 24-well microplates for experiments.

### 5.6. Cell Viability Exposed to VLPs

The cell viability due to VLPs transfection in HK2, HEK293, and HeLa cells was determined by the colorimetric CellTiter 96® AQ_ueous_ Non-Radioactive Cell Proliferation Assay (MTS, Promega Co., USA). Briefly, 2 × 10^4^ cells were grown in 96-well microplates (0.32 cm^2^, Corning Inc., USA) and incubated with VLPs for 16, 20, and 24 h, as previously described. 20 *μ*l of a 1 : 20 mixture MTS/PMS was added to 100 *μ*L/well of medium, and the cells were incubated for 90 min at 37°C. The absorbance of the formazan product was measured at 490 nm in a microplate reader ELx800 (BioTek, USA), which is directly proportional to the metabolic activity of the living cells. Measurements were performed in triplicate for each condition, and experiments were repeated at least 3 times. The results were expressed as the percent of cell viability relative to that in the negative control cell lines.

### 5.7. Transfection of VLPs-mRNA-EGFP into Cell Lines

To determine the transfection of VLPs as well as the expression of EGFP in different cell lines, 2 × 10^4^ cells were grown on 24-well plates for 24 h. VPLs containing the mRNA that codes for the EGFP were diluted at a ratio of 100 VLPs per cell with each culture medium and then were added onto the cells and incubated for 16, 20, and 24 h, as described above. A negative control was done with cells incubated without VPLs. Two additional positive control tests for the HEK293 cells were made by transfection with the pVAX1-EGFP DNA using the well-known calcium phosphate method for DNA transfection and lipofectamine reagent for RNA assay with cap, at the same conditions as that for VLPs. The cells were observed after 24 h in an inverted fluorescence microscope using 20, 40, and 60X objectives (Eclipse Ti-U Nikon, Hamamatsu 1394 ORCA-ER camera, software Imagine workbench version 6.0, Tokyo, JP).

To quantitatively corroborate the fluorescence intensity of EGFP within the cells incubated with the VLPs, 2 × 104 cells were grown in 96-well optical bottom microplates (Nunc™, Life Tech, Thermo Fisher, MA, USA). The fluorescence analysis was performed at 16 and 24 h using a 485 nm excitation wavelength and a 528 nm emission wavelength in a multimode microplate reader CYTATION 3 that uses the Gen5 data analysis software (BioTek, USA). The fluorescence emission results were expressed as the average of two independent experiments performed in triplicate.

## 6. Statistical Analysis

Data were graphed and analysed by GraphPad Prism software. The data correspond to mean ± SD of three independent experiments. Statistically significant differences were identified by one-way ANOVA, where *p* values <0.05 were considered statistically significant.

## Data Availability

The experimental data used to support the findings of this study are included within the article.
